# GLUT1 inhibitor BAY-876 induces apoptosis and enhances anti-cancer effects of bitter receptor agonists in head and neck squamous carcinoma cells

**DOI:** 10.1038/s41420-024-02106-z

**Published:** 2024-07-25

**Authors:** Zoey A. Miller, Sahil Muthuswami, Arielle Mueller, Ray Z. Ma, Sarah M. Sywanycz, Anusha Naik, Lily Huang, Robert M. Brody, Ahmed Diab, Ryan M. Carey, Robert J. Lee

**Affiliations:** 1 Department of Otorhinolaryngology, University of Pennsylvania Perelman School of Medicine, Philadelphia, PA 19104 USA; 2 Pharmacology Graduate Group, University of Pennsylvania Perelman School of Medicine, Philadelphia, PA 19104 USA; 3 Department of Physiology, University of Pennsylvania Perelman School of Medicine, Philadelphia, PA 19104 USA

**Keywords:** Head and neck cancer, Cancer metabolism, Preclinical research

## Abstract

Head and neck squamous cell carcinomas (HNSCCs) are cancers that arise in the mucosa of the upper aerodigestive tract. The five-year patient survival rate is ~50%. Treatment includes surgery, radiation, and/or chemotherapy and is associated with lasting effects even when successful in irradicating the disease. New molecular targets and therapies must be identified to improve outcomes for HNSCC patients. We recently identified bitter taste receptors (taste family 2 receptors, or T2Rs) as a novel candidate family of receptors that activate apoptosis in HNSCC cells through mitochondrial Ca^2+^ overload and depolarization. We hypothesized that targeting another component of tumor cell metabolism, namely glycolysis, may increase the efficacy of T2R-directed therapies. GLUT1 (*SLC2A1*) is a facilitated-diffusion glucose transporter expressed by many cancer cells to fuel their increased rates of glycolysis. GLUT1 is already being investigated as a possible cancer target, but studies in HNSCCs are limited. Examination of immortalized HNSCC cells, patient samples, and The Cancer Genome Atlas revealed high expression of GLUT1 and upregulation in some patient tumor samples. HNSCC cells and tumor tissue express GLUT1 on the plasma membrane and within the cytoplasm (perinuclear, likely co-localized with the Golgi apparatus). We investigated the effects of a recently developed small molecule inhibitor of GLUT1, BAY-876. This compound decreased HNSCC glucose uptake, viability, and metabolism and induced apoptosis. Moreover, BAY-876 had enhanced effects on apoptosis when combined at low concentrations with T2R bitter taste receptor agonists. Notably, BAY-876 also decreased TNFα-induced IL-8 production, indicating an additional mechanism of possible tumor-suppressive effects. Our study demonstrates that targeting GLUT1 via BAY-876 to kill HNSCC cells, particularly in combination with T2R agonists, is a potential novel treatment strategy worth exploring further in future translational studies.

## Introduction

Head and neck squamous cell carcinomas (HNSCCs) arise from the mucosa of the oral and nasal cavities, pharynx, and larynx [1], with a 5-year survival rate of 50% and often with extreme post-treatment morbidities [1]. Available first-line treatments, including surgery, radiation, and chemotherapy, often result in cosmetic deformities, speech dysfunction, dysphagia, and feeding tube or tracheostomy dependence [2, 3]. There are currently no first-line targeted therapies with proven efficacy for primary treatment of HNSCC without distant metastasis, and targeted therapies have marginal benefit for recurrent and metastatic tumors [4].

We recently identified bitter taste receptors (taste family 2 receptors, or T2Rs) as novel targets to activate apoptosis in HNSCC. T2R activation with bitter agonists in HNSCC cells cause mitochondrial Ca^2+^ overload and depolarization, reducing NADH production and activating apoptosis [5, 6]. Increased expression of T2R isoforms T2R4 and T2R14 are positively correlated with HNSCC survival. Many clinical drugs are bitter and might be repurposed to target tumor-expressed T2Rs [7]. This may be particularly efficacious in HPV-associated HNSCC, which is associated with increased T2R14 tumor expression [5, 6]. We recently showed that lidocaine, a local anesthetic already approved for use in HNSCC surgeries, activates T2R14 and downstream apoptosis in HNSCC cells [6]. Interestingly, a recent multicenter randomized controlled clinical trial demonstrated that breast cancer patients receiving peritumoral injection of 1% lidocaine prior to surgery had greater disease-free and overall survival compared with uninjected patients [8, 9]. As breast cancer cells also express T2R14 [10], this survival benefit may be due to T2R14 activation, further supporting a potential role for T2R agonists as therapies for solid tumors. Understanding how to enhance the efficacy and potency of T2R agonists is important for maximizing the potential benefits of such a therapy.

We hypothesized that simultaneously targeting another major component of tumor cell metabolism, glycolysis, may increase the efficacy and potency of T2R agonists. Glucose from dietary carbohydrates and gluconeogenesis is a primary cellular energy source [11] transported into cells via glucose transporters (GLUTs; *SLC2A* family) [12]. Fourteen GLUT proteins exist in humans (GLUT1-14) [13]. Changes in function or expression of GLUTs can alter cell metabolism and contribute to Alzheimer’s, diabetes, heart disease, and cancer [14–18]. Cancer cells can alter metabolism to promote proliferation, [19] often enhancing glucose uptake [20] and shifting towards anaerobic glycolysis and lactate production. This is known as the Warburg effect [21].

GLUT1 is expressed in many cancers [14–16, 19], and inhibiting GLUT1 is a potential anti-glycolytic strategy for cancer treatment [22]. Solid tumors with higher GLUT1 expression are associated with decreased patient survival [23]. Downregulating or inhibiting GLUT1 in vitro halts proliferation of breast and prostate cancer cells [24, 25]. GLUT1 overexpression and knockdown may enhance and reduce proliferation, respectively, in HNSCC in vitro and xenograft models [26, 27]. However, localization and pharmacological inhibition of GLUT1 have not been explored in HNSCC [28]. A highly selective GLUT1 inhibitor, BAY-876, was recently identified but not yet widely tested in vitro [29]. We hypothesized that inhibition of GLUT1 with BAY-876 may have anti-proliferative effects in HNSCC cells and may enhance the efficacy and/or potency of T2R agonists.

Here, we show that HNSCC cell lines and patient samples express GLUT1, possibly with higher expression in tumor vs normal tissue. GLUT1 in these samples is localized on the plasma membrane and within the cytoplasm. Inhibition of GLUT1 with BAY-876 blocked cancer cell glucose uptake, induced apoptosis, and reduced IL-8 production. Moreover, GLUT1 inhibition enhanced apoptotic effects observed with T2R agonists. GLUT1 could thus serve as a novel therapeutic target in HNSCCs.

## Results

### GLUT1 Is Expressed in HSNCC Cell Lines and Tumors

**Fig. 1:**
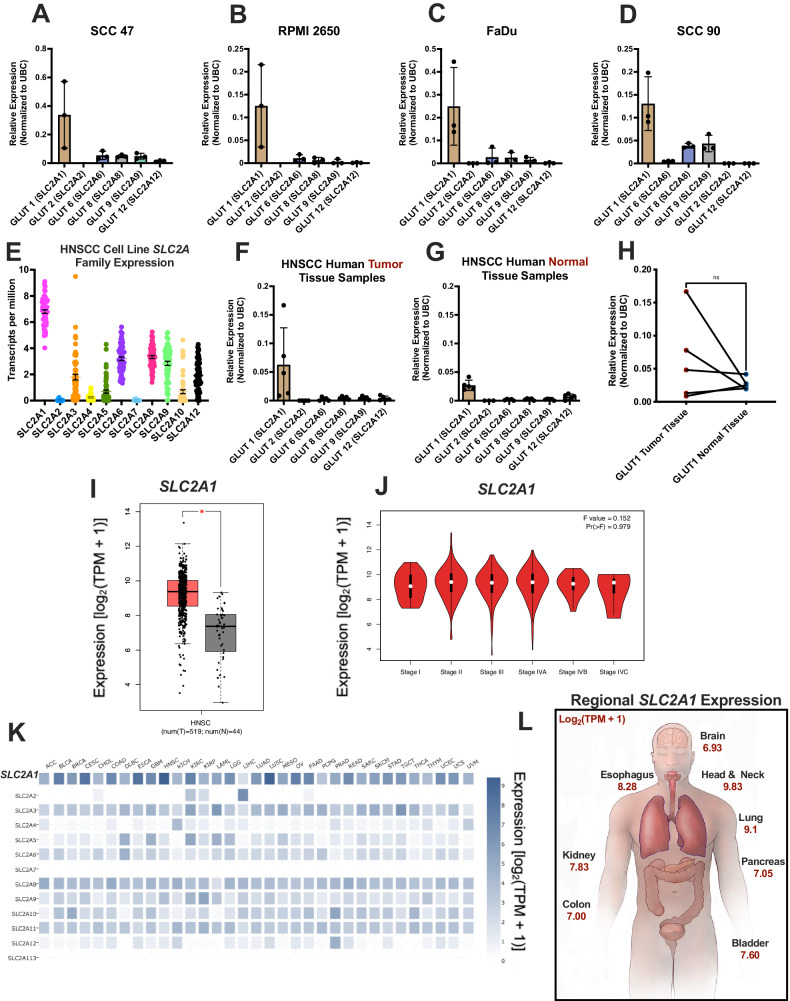
HNSCC cell lines and patient samples express *SLC2A1* (GLUT1). mRNA expression of several *SLC2A* transporters in HNSCC cell lines **A** SCC47, **B** RPMI2650, **C** FaDu, and **D** SCC90 measured using qPCR relative to housekeeping UBC (mean ± SD, n = 3, separate passages of each cell type). **E** *SLC2A* family expression in 62 total HNSCC immortalized cell lines downloaded from Cancer Dependency Map (depmap) Project (Broad Institute). mRNA expression of several *SLC2A* transporters in HNSCC **F** tumor and **G** normal tissue relative to UBC (mean ± SD with n = 6 patients). **H** Matched expression of *SLC2A1* between HNSCC patient tumors and normal tissue relative to UBC (mean ± SD; n = 6 patients). No significant difference by paired t-test. P < 0.05 (*), P < 0.01 (**), P < 0.001 (***), and no statistical significance (ns or no indication). **I** Boxplot of *SLC2A1* expression in HNSCC tumor samples (n = 519) and normal tissue samples (n = 44), generated using GEPIA2. P < 0.05 (*). **J** Plots of *SCL2A1* expression showing no marked changes with cancer stage (bottom), generated using GEPAI2. **K** Heatmap of *SLC2A1* expression across multiple cancer types from GEPIA2, showing a pattern of *SLC2A1* being the highest expressed or among the highest expressed *SLC2A* isoforms across cancer types. **L** Diagram of internal regions with highest upregulation of *SLC2A1* in tumor tissue (regardless of cancer sub-type). Adapted from GEPIA2 *Interactive Bodymap*. TCGA cancer abbreviations: ACC adrenocortical carcinoma, BLCA bladder urothelial carcinoma, BRCA breast invasive carcinoma, CESC cervical squamous cell carcinoma and endocervical adenocarcinoma, CHOL cholangio carcinoma, COAD colon adenocarcinoma, DLBC lymphoid neoplasm diffuse large B-cell lymphoma, ESCA esophageal carcinoma, GBM glioblastoma multiforme, HNSC head and neck squamous cell carcinoma, KICH kidney chromophobe, KIRC kidney renal clear cell carcinoma, KIRP kidney renal papillary cell carcinoma, LAML acute myeloid leukemia, LGG brain lower grade glioma, LIHC liver hepatocellular carcinoma, LUAD lung adenocarcinoma, LUSC lung squamous cell carcinoma, MESO mesothelioma, OV ovarian serous cystadenocarcinoma, PAAD pancreatic adenocarcinoma, PCPG pheochromocytoma and paraganglioma, PRAD prostate adenocarcinoma, READ rectum adenocarcinoma, SARC sarcoma, SKCM skin cutaneous melanoma, STAD stomach adenocarcinoma, TGCT testicular germ cell tumors, THCA thyroid carcinoma, THYM thymoma, UCEC uterine corpus endometrial carcinoma, UCS uterine carcinosarcoma, UVM uveal melanoma.

We first measured GLUT1 expression in human HNSCC cell lines. HNSCC cell lines SCC47, FaDu, RPMI2650, and SCC90 expressed several GLUTs (Fig. 1A–E). GLUT1 was the highest expressed in all cell lines (Fig. 1A–E). In addition, GLUT1 was the highest isoform in both tumor and corresponding contralateral normal tissue from 6 patients (Fig. 1F–H, Supplementary Fig. 1). Some patients had higher GLUT1 in tumor tissue compared to their normal tissue (Fig. 1H), though aggregate results were not significantly different in this small data set.

We further explored GLUT1 expression in HNSCC in a larger dataset, The Cancer Genome Atlas (TCGA), using GEPIA2 [30]. GLUT1/*SLC2A1* was significantly upregulated in HNSCC tumors (Fig. 1I, J). GLUT6/*SLC2A6* was also upregulated in HNSCC tumors, revealing a possible additional transporter of interest (Supplementary Fig. 2). BAY-876 may have the potential to act as a targeted therapy due to increased GLUT1 in tumor tissue, alone or combined with bitter agonists. As HNSCCs are among cancers with highest expression of GLUT1/*SLC2A1* (Fig. 1K, L), BAY-876 may have enhanced efficacy in HNSCC.

### GLUT1 is localized on the cell membrane and in perinuclear regions

**Fig. 2:**
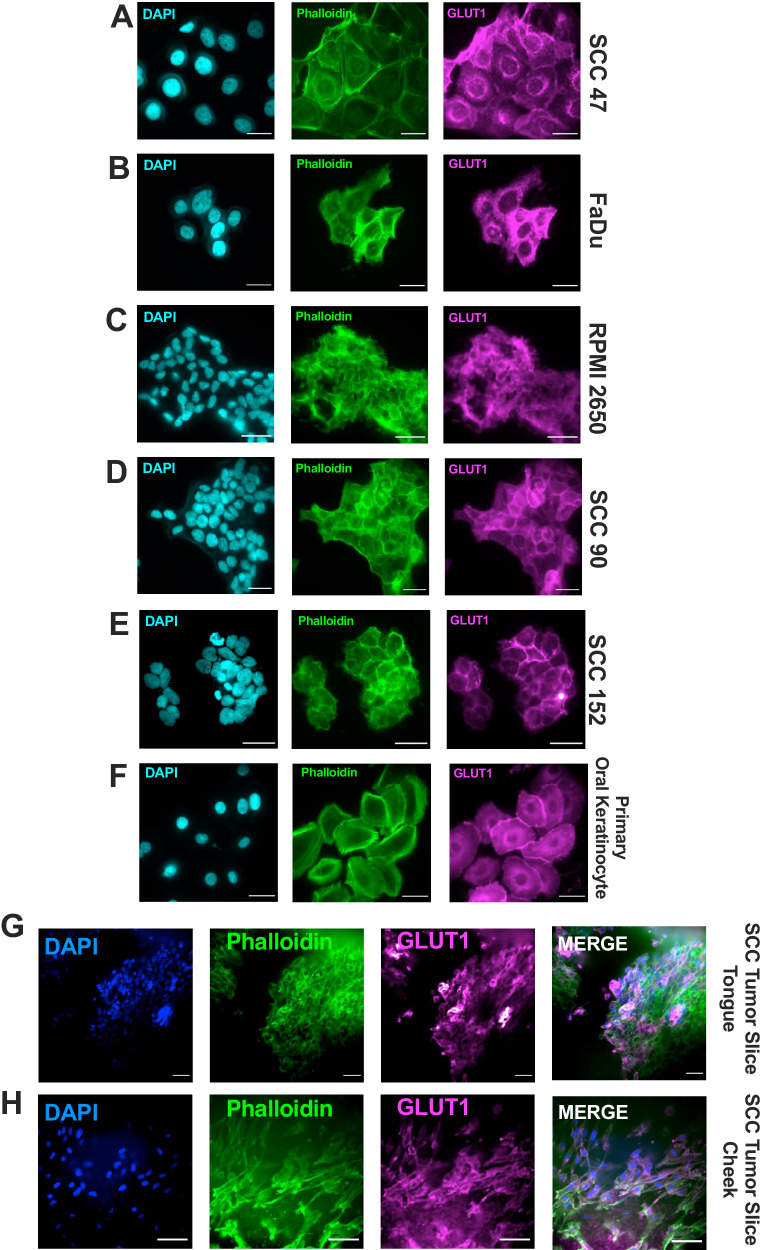
HNSCC cells express GLUT1 on the plasma membrane and perinuclear regions. GLUT1 (Cell Signaling mAB #71831 or mAB #12939) expression in **A** SCC47, **B** FaDu, **C** RPMI2650, **D** SCC90, **E** SCC152, and **F** primary oral keratinocytes via immunofluorescence with DAPI (cyan, nucleus) and phalloidin (green, actin) markers. GLUT1 expression in **G** oral tongue or **H** buccal cheek SCC tumor slice from patient surgical resection. Scale bars = 30 μm. Images represent phenotype observed in n > 12 separate cultures.

In normal tissues, GLUT1 on the cell membrane transports extracellular glucose into the cell [31]. We found that GLUT1 is expressed both in perinuclear regions and on the plasma membrane (Fig. 2A–E) in HNSCC cells. While some cells (SCC47 and FaDu) exhibited strong perinuclear staining, others (SCC90, SCC152, RPMI2650) did not (Fig. 2A–E; Supplementary Fig. 3A, B). Oral keratinocytes, a non-cancerous primary precursor of HNSCCs, also expressed GLUT1 on the membrane and intracellularly (Fig. 2F). SCC tumor slices (collected from oral cavity tumor resections) revealed primarily plasma membrane GLUT1 localization (Fig. 2G, H). The heterogeneity of localization between HNSCC cells lines was unexpected as GLUT1 is frequently expressed on the plasma membrane for the purpose of efficient uptake of extracellular glucose.

**Fig. 3:**
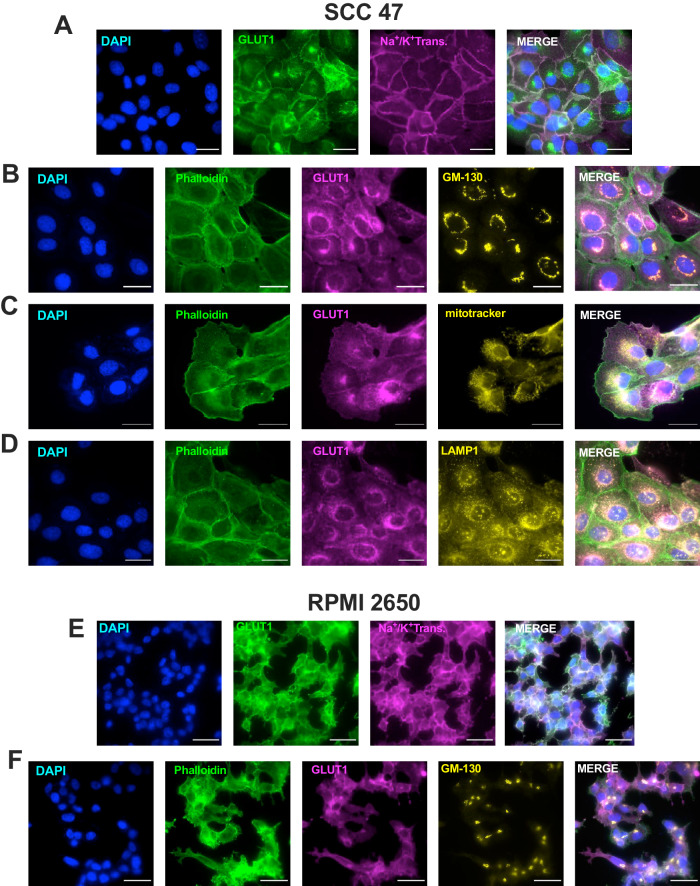
GLUT1 differentially localizes to the cell membrane and Golgi in HNSCC cell lines. GLUT1 (Cell Signaling mAB #71831 or mAB #12939)and **A** Na^+^/K^+^-ATPase pump (cell membrane; Abcam #ab76030), **B** GM130 (Golgi apparatus; BD Transduction Laboratories #610822), **C** mitotracker (mitochondria; Thermo Fisher #M22425), or **D** LAMP1 (lysosome; Abcam #ab24170) expression in SCC47. GLUT1 and **E** Na^+^/K^+^-ATPase pump (cell membrane; Abcam #ab76030) or **F** GM130 (Golgi apparatus; BD Transduction Laboratories #610822) expression in RPMI2650 cells via immunofluorescence with DAPI (cyan, nucleus) and phalloidin (green, actin) markers. Scale bars = 30 μm. Images best represent phenotype observed in n > 12 separate cultures.

To understand if the perinuclear-expressed GLUT1 observed in some cells localized to a specific organelle, SCC47 cells were co-stained with markers for plasma membrane (Na^+^/K^+^ pump), Golgi (GM-130), mitochondria (Mito tracker), and lysosome (LAMP1) (Fig. 3A–D). GLUT1 appeared to co-localize largely to the Golgi (Fig. 3B). We tested if perinuclear localization of GLUT1 depended on extracellular glucose, as GLUT1 may translocate to the plasma membrane in response to higher glucose levels [32]. However, when glucose (DMEM with 4.5 g/L glucose) was replenished 3 h before imaging, GLUT1 remained perinuclear in SCC47 cells (Supplementary Fig. 3C). In contrast to SCC47 cells, RPMI 2650 cells showed validated plasma membrane GLUT1 localization with the use of Na^+^/K^+^ pump marker. GM-130 did not co-localize to GLUT1 in RPMI2650 cells. These findings reveal that GLUT1 is expressed on both the cell membrane and Golgi to varying degrees in HNSCC cells.

### BAY-876 inhibits glucose uptake

We hypothesized that GLUT1 inhibition may hinder glycolysis, the major non-mitochondrial energy-producing pathway in cancer cells, by limiting glucose availability. While there are well-documented GLUT1 inhibitors, including STF-31, WZB-177, and apigenin [33–35], they are not specific to GLUT1. The more recently identified BAY-876 has a 250-500x higher affinity for GLUT1 (IC_50_ ~ 0.002 μM) compared to other inhibitors [29]. We hypothesized that BAY-876 would inhibit glucose uptake in HNSCC cells, which may have proapoptotic effects alone or in combination with T2R agonists that depolarize mitochondria.

**Fig. 4:**
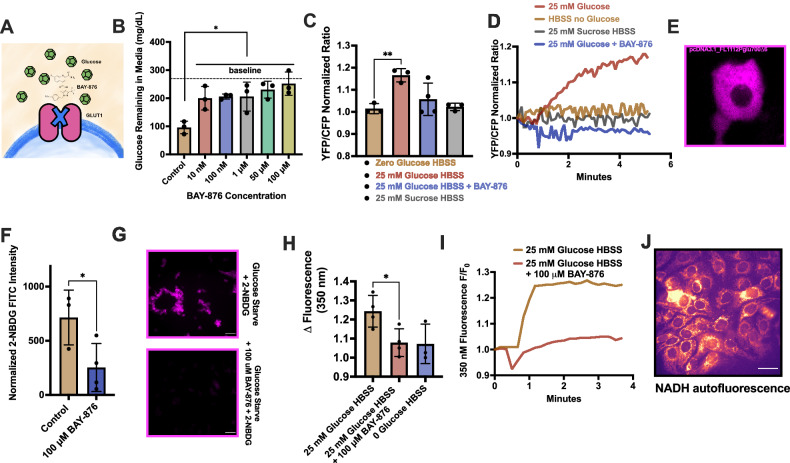
BAY-876 inhibits glucose uptake in HNSCC cells. **A** Representative visual of drug action of BAY-876. **B** Extracellular SCC47 media glucose concentration following 24-h treatment with 0–100 µM BAY-876. Dotted line represents basal glucose level in cell media at 0 h (DMEM, high glucose); means ± SD with n = 3 of separate cultures of cells. Significance by 1-way ANOVA with Bonferroni’s posttest comparing each BAY-876 concentration to control. **C**–**E** Glucose uptake over 5 min with the addition of 25 mM glucose +/- BAY-876 or sucrose at ~45 s in SCC47 cells. Both **C** peak uptake and **D** uptake over time were measured with **E** FRET (YFP/CFP) biosensor, FL1112Pglu700Δ6. Sucrose used as negative control. Fluorescence means ± SD with n = 3 of separate cultures of cells. Significance by 1-way ANOVA with Bonferroni’s posttest comparing each condition to control. **F**, **G** 2-NBDG uptake after 15 min with 1 h prior incubation ± BAY-876. Fluorescence intensity represents amount of 2-NBDG taken up by the cell. Fluorescent mean ± SD with n = 3 of separate cultures of cells. Significance unpaired t-test. Autofluorescence of NADH at **H** peak and **I** over time with the addition of 25 mM glucose +/- BAY-876 and **J** representative image of NADH autofluorescence (ex: 340 nM; em: 475 nM). Fluorescence means ± SD with n = 3 of separate cultures of cells. Significance by 1-way ANOVA with Bonferroni’s posttest. P < 0.05 (*) P < 0.01 (**), P < 0.001 (***), and no statistical significance (ns or no indication).

BAY-876 (10 nM to 100 µM) inhibited glucose uptake from SCC47 media over 24 h (Fig. 4A, B). Additionally, FLII12Pglu-700u,δ6, a glucose-sensing biosensor, was used to measure glucose uptake in real time via live cell imaging [36]. BAY-876 blocked glucose uptake during addition of 25 mM glucose (Fig. 4C–E). BAY-876 also inhibited uptake of fluorescent glucose analog 2-NBDG [37] (Fig. 4F, G). Thus, BAY-876 reduces glucose uptake consistent with GLUT1 inhibition. NADH is produced during glycolysis [38]. By imaging NADH autofluorescence [39], we found that BAY-876 blocked increased production of NADH with the addition of 25 mM glucose in SCC47 cells (Fig. 4H–J), indicating that BAY-876 inhibition of GLUT1 reduces downstream glycolysis by limiting glucose availability.

### BAY-876 reduces cell viability

**Fig. 5:**
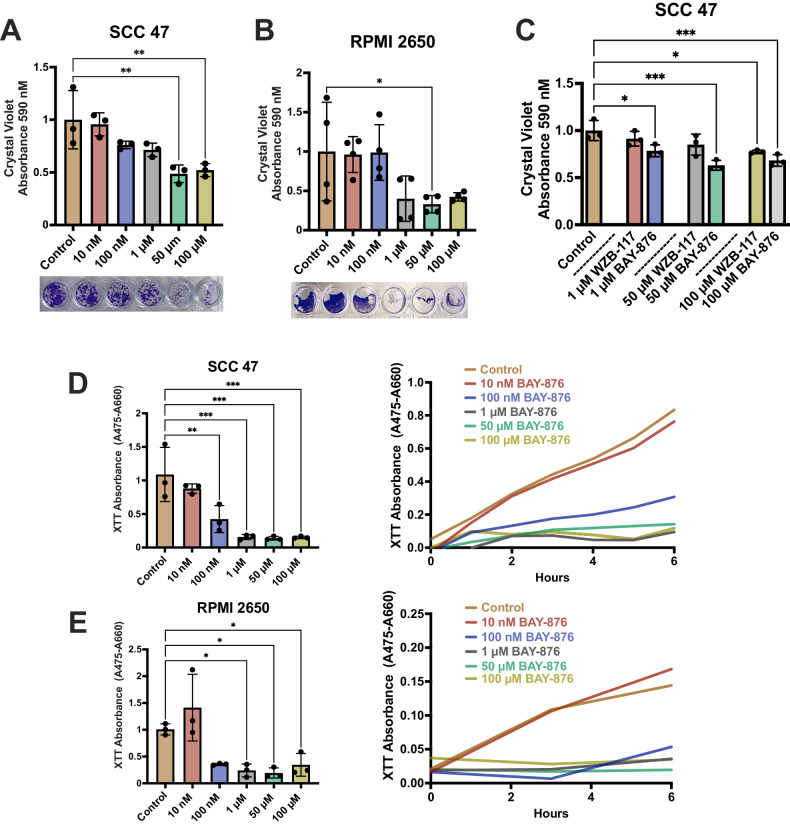
BAY-876 decreases cell viability and metabolism. **A, B** Crystal violet absorbance and representative images showing live adherent cells following treatment with 0-100 μM BAY-876 after 24 h in **A** SCC47 and **B** RPMI2650 cells. Absorbance means ${\boldsymbol{\pm }}\,$SD with n > 3 separate cell passages. Significance by 1-way ANOVA with Bonferroni’s posttest comparing BAY-876 to control. **C** Crystal violet absorbance showing live adherent cells following treatment with BAY-876 or WZB-117 after 24 h. Absorbance means ${\boldsymbol{\pm }}\,$SD with n = 3 separate cell passages. Significance by 1-way ANOVA with Bonferroni’s posttest comparing BAY-876 to control. Absorbance values of NADH production (via addition of XTT dye) at 6 h (left) and over time (right) in **D** SCC47 and **E** RPMI2650 cells. Less absorbance (475–660 nm) indicates reduced NADH production; means ${\boldsymbol{\pm }}\,$SD with n = 3 separate cell passages. Significance by 1-way ANOVA with Bonferroni’s posttest comparing BAY-876 to control. P < 0.05 (*), P < 0.01 (**), P < 0.001 (***), and no statistical significance (ns or no indication).

GLUT1 inhibition may decrease cancer cell viability by reducing energy supplies [40]. Crystal violet was used to measure viable cells following treatment with BAY-876 [41]. BAY-876 reduced viable SCC47, RPMI2650, and FaDu cells after 24 h (Fig. 5A, B; Supplementary Figs. 4A, B). We compared BAY-876 to WZB-117, another GLUT1 inhibitor, in SCC47 cells [42]. BAY-876 reduced viability at concentrations as low as 1 μM; whereas, WZB-117 inhibited viability at 100 uM, suggesting that BAY-876 is more potent (Fig. 5C). BAY-876 also inhibited viability in primary oral keratinocytes (Supplementary Fig. 4C), suggesting it may have a narrow therapeutic window or may work best in combination with other cancer cell-specific drugs.

We measured cell metabolism using XTT, which also measures NADH production [43]. BAY-876 reduced cell metabolism over only 6 h in SCC47, RPMI2650, and FaDu cells (Fig. 5E, F; Supplementary Fig. 4D). The mitochondria produces the bulk of intracellular NADH [44]. From our XTT results, we expected that the mitochondrial may be depolarized with inhibition of GLUT1. Surprisingly, we found that BAY-876 hyperpolarized mitochondria in SCC47 and RPMI2650 cells. BAY-876 was previously shown to increase mitochondrial dependence in T cells and macrophages [45]. Our findings support a similar metabolic shift from glycolysis towards other types of mitochondrial ATP production in HNSCC cells (Supplementary Fig. 5).

### BAY-876 induces apoptosis at higher concentrations

**Fig. 6:**
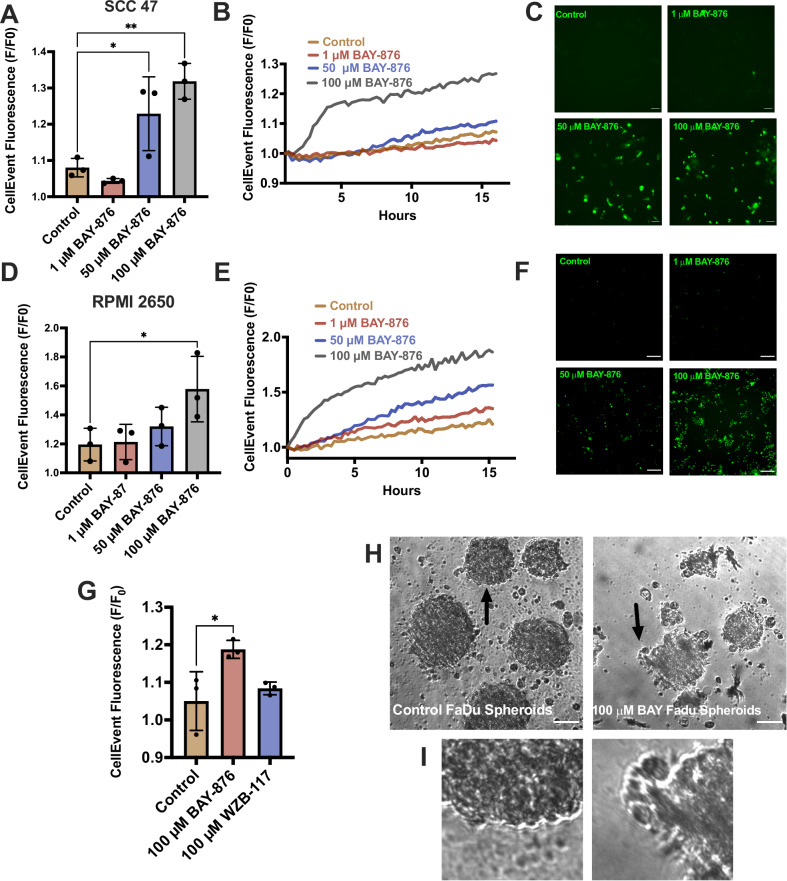
BAY-866 induces apoptosis in HNSCC cells. Fluorescence values and images of CellEvent (increase indicative of caspase 3/7 activation) at 15 h (left bar graph and representative images) and over time (middle xy plot) of **A**–**C** SCC47 and **D**–**F** RPMI2650 cells treated with BAY-876. Fluorescence means ${\boldsymbol{\pm }}\,$SD with n = 3 separate cell passages. Significance by 1-way ANOVA with Bonferroni’s posttest comparing BAY-876 to control. **G** Fluorescence values of CellEvent at 15 h of SCC47 cells treated with BAY-876 or WZB-117; means ${\boldsymbol{\pm }}\,$SD with n = 3 separate cell passages. Significance by 1-way ANOVA with Bonferroni’s posttest comparing BAY-876 and WZB-117 to control. **H**, **I** FaDu spheroids 5 days-post low attachment plating. Cells treated ± BAY-876 for 24 h. DIC images (acquired after 24 h) show **H** whole representative spheroid and **I** a close-up of the edge of the spheroid. Scale bars = 30 µm. Images best represent phenotype observed in n > 12 separate cultures. P < 0.05 (*), P < 0.01 (**), P < 0.001 (***), and no statistical significance (ns or no indication).

GLUT1 inhibition induces apoptosis in some cancers [46], but there are limited studies of GLUT1 in HNSCC [47] and none with BAY-876. To test if BAY-876 induces apoptosis, HNSCC cells were treated with BAY-876 over 15 h with fluorescent CellEvent dye to measure caspase-3 and/or -7 cleavage [48]. BAY-876 induced apoptosis in SCC47, FaDu, and RPMI2650 cells (Fig. 6A–F; Supplementary Fig. 6A–C). While WZB-117 inhibited viability (noted above), it did not induce apoptosis at comparable concentrations (Fig. 6G). The effects of BAY-876 on a 3D tumor model were evaluated using FaDu spheroids [49]. After 24 h, 100 µM BAY-876-treated spheroids showed reduced edge integrity (Fig. 6H, I), possibly signifying apoptosis. However, comparison of apoptotic rates in oral primary keratinocytes and HNSCC cells exposed to BAY-876 showed no significant differences over 15 h (Supplementary Figs. 6D, E). We nonetheless hypothesized that BAY-876 inhibition of glucose transport and glycolysis may have synergistic effects with T2R agonists, which reduce mitochondrial metabolism specifically in cancer cells [5, 6].

### BAY-876 enhances the effects of T2R agonists denatonium benzoate and lidocaine

**Fig. 7:**
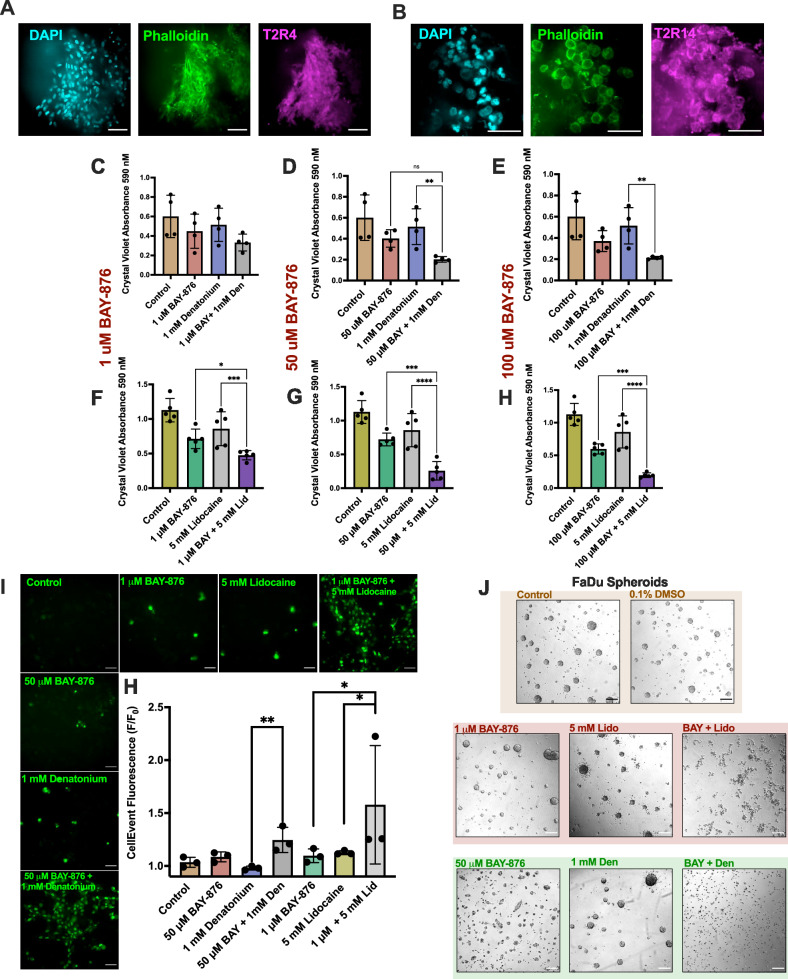
BAY-876 enhances the ability of bitter agonists to kill HNSCC cells. **A** T2R4 (Thermo Fisher PA-67752 antibody) or **B** T2R14 (LS-Bio T2R14/TRB1 C386266 antibody) expression in cheek SCC tumor slice. Images best represent phenotype observed in n = 1 patient. **C–H** Crystal violet absorbance representing live/adherent SCC47 cells following treatment with 1–100 μM BAY-876± **C**–**E** 5 mM Lidocaine or **F**–**H** 1 mM denatonium after 24 h; means ${\boldsymbol{\pm }}\,$SD with n > 3 separate cell passages. Significance by 1-way ANOVA with Bonferroni’s posttest comparing BAY-876 and lidocaine or denatonium treatments alone to BAY-876 + lidocaine or denatonium. **I** Fluorescence values and images of CellEvent caspase-3 and -7 dye (increase in fluorescence indicative of caspase activation) at 15 h (bar graph and representative images) in SCC47 cells treated with BAY-876 ± lidocaine or denatonium. Scale bars 25 μm. Concentrations used were determined from the lowest BAY-876 concentration that significantly reduced proliferation with bitter agonists. Fluorescence means ${\boldsymbol{\pm }}\,$SD with n = 3 separate cell passages. Significance determined by 1-way ANOVA with Bonferroni’s posttest comparing BAY-876 and lidocaine/denatonium alone to BAY-876 with lidocaine/denatonium. **J** DIC images of FaDu cell line spheroid formation over 24 h with BAY-876 ± lidocaine or denatonium. Images are representative of 3 separate cultures for each condition. Scale bars 200 μm. P < 0.05 (*), P < 0.01 (**), P < 0.001 (***), and no statistical significance (ns or no indication).

Co-administration of therapeutics targeting multiple independent mechanisms [50] can produce a combined effect greater than each drug alone, often at lower concentrations [51]. It is unknown if BAY-876 enhances other pro-apoptotic therapeutics, or vice-versa. HNSCCs express T2R bitter taste receptors T2R4 and T2R14 (shown in SCC patient tumor slices, Fig. 7A, B) and higher expression is favorably associated with patient survival [5]. While some patients have elevated expression of T2R transcripts in their tumors compared to normal tissue [5], tumor cells have dramatically higher levels of T2R protein resulting in dramatically higher T2R responses [6]. Activation of T2R4 and 14 by bitter agonists induces apoptosis in HNSCCs (model shown in Supplementary Fig. 7), while normal primary keratinocytes do not die [5, 6]. T2R-driven apoptosis occurs via mitochondrial Ca^2+^ overload and downstream proteasome inhibition.

We tested if BAY-876 has enhanced efficacy with T2R agonists lidocaine and/or denatonium benzoate to kill HNSCC cells more effectively. Using crystal violet to measure viable cells, we found that 50 µM BAY-876 enhanced effects of 1 mM denatonium benzoate (Fig. 7C–E) while 1 µM BAY-876 enhanced effects of 5 mM lidocaine (Fig. 7F–H). We previously determined that the effective apoptotic concentrations of denatonium benzoate and lidocaine are 15 and 10 mM, respectively [5, 6]. Strikingly, lower sub-apoptotic concentrations of BAY-876 plus lidocaine or denatonium benzoate induced apoptosis in HNSCC cells over 15 h and inhibited spheroid formation over 24 h (Fig. 7I, J). Thus, BAY-876 could potentially be utilized in conjunction with bitter agonists to target tumor metabolism through dual mechanisms at concentrations of BAY-876 where normal surrounding keratinocytes do not undergo apoptosis.

### BAY-876 reduces TNF-alpha-induced production of IL-8

**Fig. 8:**
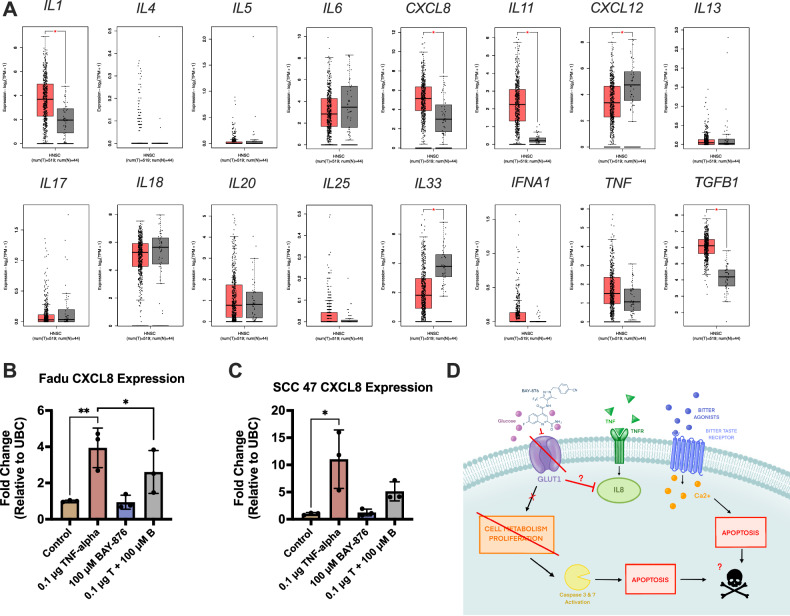
IL-8 expression is elevated in HNSCC and BAY-876 reduces TNF-alpha-induced IL-8. **A** Box plots of pro-inflammatory cytokines expression, showing *CXCL8* to be amongst the highest expressed cytokines in HNSCC tumor samples (n = 519) and normal tissue samples (n = 44). Generated using GEPIA2. RNA fold change of *CXCL8* in **B** FaDu and **C** SCC47 cells after 24-h treatment with TNF-alpha (known IL-8 inducer) ± BAY-876. Fold change is relative to control (untreated). Fold change means ± SD with n = 3 separate passages of cells. 1-way ANOVA with Bonferroni’s posttest comparing Control to TNF-alpha treatment alone and TNF-alpha treatment alone to TNF-alpha + BAY-876. P < 0.05 (*), P < 0.01 (**), P < 0.05 (*), P < 0.01 (**), P < 0.001 (***), and no statistical significance (ns or no indication). TCGA cancer abbreviations shown in Fig. 1 legend.

Cancer cells and immune cells in the tumor microenvironment produce cytokines and chemokines [52] to influence tumorigenesis [53]. Certain types of inflammation create a tumor-suppressive environment [54], but inflammation can also fuel hypoxia, support vasculature, and alter extracellular matrix, creating a tumor-promoting environment [55]. Because of this, pro-inflammatory cytokines/chemokines within the tumor microenvironment may be targets to augment tumor inflammation [52, 56]. We measured expression of inflammatory cytokines and chemokines in HNSCCs using TCGA, uncovering elevated interleukin-8 (IL-8, *CXCL8*) expression in HNSCC tumors. Tumor necrosis factor-alpha (TNF-α) is a known inducer of IL-8, but TNF-α expression was not elevated in HNSCC tumors (Fig. 8A) [57]. Increased cellular glucose uptake may promote inflammatory cytokine production [58]. BAY-876 reduced TNF-α-stimulation of IL-8 in HNSCC cells (Fig. 8B, C). This offers novel insight into mechanisms of how BAY-876 may influence tumor microenvironment.

## Discussion

GLUT1 inhibitors target cancer cell glycolysis [59]. Elevated GLUT1 expression in tumors presents an opportunity for targeted inhibition [60]. This paradigm may be useful for HNSCC, as current therapies due not overcome poor survival and are not cancer cell-specific [61] with significant morbidity [62]. We show that HNSCC cells and tumors express GLUT1 (*SLC2A1*), and TCGA suggests GLUT1 is upregulated in HNSCC tumors. As GLUT1 may be a biomarker in HNSCCs [63], tumor GLUT1 expression could be used for both prognosis and treatment selection. During the typical multi-week delay between biopsy and definitive treatment [64], HNSCC tumors could be evaluated for GLUT1, presenting an opportunity for personalized medicine.

We found that HNSCC cells have largely membrane or cytoplasmic (perinuclear) localization of GLUT. This was an unexpected result. We believe that the perinuclear localization is specific to the Golgi apparatus, which glycosylates secreted proteins [65]. This requires the uptake of glucose into the organelle [65], which may partly explain why GLUT1 is localized to the Golgi. Cancer cells can have altered glycosylation activity and inhibition of enzymes within this process have been successful at decreasing cancer growth and progression [66]. In addition to inhibiting extracellular glucose uptake, BAY-876 might thus also disrupt GLUT1 function and glycosylation in the Golgi [67]. This localization pattern is similarly documented in prostate carcinoma cells, indicating this potential mechanism could be relevant to other cancers [68]. However, more work is needed to understand why only some HNSCC cell lines express noticeable GLUT1 in the Golgi.

Nonetheless, BAY-876, a novel high-affinity GLUT1 inhibitor [29], inhibits glucose uptake and viability in HNSCC cells independent of GLUT1 Golgi vs plasma membrane localization. Depletion of glucose uptake inhibits glycolysis, leading to decreased cell viability and metabolism. However, we surprisingly found that the mitochondria does not depolarize during BAY-876 treatment. In fact, there is a significant hyperpolarization of the organelle. This could be due to a shift in dependence on mitochondrial non-glucose metabolism for energy due to lack of glucose supply, still causing organelle stress. An increase in mitochondrial hyperpolarization can be linked with early apoptotic events, as observed with BAY-876 [69]. Although GLUT1 transcriptional regulation has been widely studied in HNSCCs specifically, downstream action of GLUT1 pharmacological inhibition is not well defined. Our findings with BAY-876 reveal a novel mechanism of GLUT1 inhibition-induced apoptosis in HNSCC cells. A summary of the effects of BAY-876 observed in this study are shown in Supplementary Table 1.

We previously found that activation of T2R isoforms T2R4 and T2R14 induce apoptosis in HNSCC cells through a Ca^2+^ dependent mechanism, [5, 6]. Like GLUT1, T2R4 and T2R14 expression and function are elevated in HNSCC tumors [5]. Lidocaine and denatonium benzoate (agonists of T2R4 and T2R14, respectively [6]) have an enhanced effect with BAY-876 to inhibit viability and induce apoptosis. This could be through increased mitochondrial damage with both compounds. Reducing the necessary concentrations of each compound in a combination therapy may decrease toxicity to non-malignant cells. Although sensitization to traditional chemotherapies with bitter agonists have been explored, the combination with a GLUT1 inhibitor, such as BAY-876, is novel [70]. Beyond bitter agonists, combinatory effects between BAY-876 and any other existing therapies are also not documented.

Many cancers can evade or become resistant to a singular chemotherapy [70]. Administering a dual therapy that acts through independent cellular pathways increases the chance of successful cancer cell death without evasion [71]. This is also relevant as tumor energy needs are heterogenous between cells, which could dictate how individual cells respond to glycolytic targeting therapies [72]. Adding bitter agonists to this regiment could ensure total cell death independent of glycolytic status. Dual therapy approaches are actively being pursued in HNSCC research, making our findings highly relevant [73]. Genetic inhibition of GLUT1 can chemosensitize HNSCC cells to cisplatin [28]. Our work suggests a pharmacological approach of combining BAY-876 with bitter agonists or possibly other chemotherapies could maximize the efficacy of GLUT1 targeting in HNSCC.

HNSCCs often affect accessible mucosal regions, allowing therapies to be delivered in a localized fashion. In HNSCC, BAY-876 and/or T2R agonists could be delivered as a topical irrigation or local injection independently or in conjunction with an existing treatment, including at the time of surgery or during radiation therapy. This localized route of administration is currently being explored in HNSCCs with both immunotherapies and traditional chemotherapies [74]. While these studies are limited, our work may prompt the expansion of non-systemic, localized therapy.

Cytokines and chemokines in the tumor microenvironment can promote inflammation that enhances tumorigenesis [75]. We showed that BAY-876 reduces TNF-α-induced IL-8 production. Elevated IL-8 promotes cancer cell evasion from the effects of chemo and immunotherapies in gastric cancer [76]. In HNSCCs, elevated IL-8 correlated with poorer response to radiation [77]. Reducing IL-8 could create a favorable tumor microenvironment for HNSCC clearance. One possible mechanism behind the IL-8 alterations could be the reduction of lactate production [78], which activates in HIF1α and NF-κβ in tumor endothelial cells [79]. Further work is needed in HNSCC to define interactions between GLUT1 and cell and tumor inflammation.

One limitation of our study is the lack of an in vivo model. Modeling T2R function in animals is difficult due to differing numbers of *TAS2R* gene isoforms (e.g., 35 in mouse vs 25 or 26 in humans) and a lack of correlation of evolutionarily orthologous human and mouse isoforms (based on sequence) with their activating agonists [80]. However, *SLC2A1* is orthologous from humans to mice. Testing the effects of BAY-876 in-vivo are thus a necessary future direction. Combination therapy with bitter agonists could also be studied through use of human tumor xenograft mouse models and/or histology studies of human tumor samples. While our GLUT1 expression data from online databases encompasses a large population, our own GLUT1 expression measurements from HNSCC patients (n = 6) is limited and does not support strong conclusions. We would like to expand this data set in the future. In addition, we aim to elucidate the mechanism between GLUT1 inhibition and IL-8 reduction and explore additional cytokine players.

Taken together, we reveal that GLUT1 could serve as a therapeutic target in HNSCCs. BAY-876 efficaciously inhibits GLUT1, leading to apoptosis and can reduce TNF-α-induced IL-8 production. Addition of bitter agonists enhances the apoptotic effects of BAY-876, revealing a potential dual therapy for HNSCCs. GLUT1 inhibition with BAY-876 and T2R activation with bitter agonists shows promise at beneficially modulating multiple parameters of HNSCC tumor cell physiology.

## Methods

Detailed experimental methods for live cell imaging, glucose uptake, other metabolic measurements, immunofluorescence, and qPCR can be found in the Supplementary Methods section of the Supplementary Material. All reagents used and their source/supplier information can be found in Supplementary Table 2. Pharmacological agonists and inhibitors and their targets are shown in Supplementary Table 3.

### Cell culture

SCC4 (ATCC CRL-1624), SCC47 (UM-SCC-47; Millipore SCC071), SCC90 (ATCC CRL-3239), FaDu (ATCC HTB-43), RPMI2650 (ATCC CCL-30), SCC152 (ATCC CRL-3240), and Primary Gingival Keratinocytes (PCS-200-014) cell lines were from ATCC (Manassas, VA, USA) or MilliporeSigma (St. Louis, MO USA), verified by STR sequencing, and tested for mycoplasma at least monthly. Cells were grown in high glucose Dulbecco’s modified Eagle’s medium (Corning; Glendale, AZ, USA) with 10% FBS (Genesee Scientific; El Cajon, CA, USA), penicillin/streptomycin mix (Gibco; Gaithersburg, MD, USA), and nonessential amino acids (Gibco) or Dermal Cell Basal Medium (PCS-200-030) with Keratinocyte Growth Kit (PCS-200-040; ATCC). 0.25% EDTA Trypsin (Gibco, Gaithersburg, MD, USA) was used at 37 °C for 10 min for single-cell suspension solution for experimental plating. Cells were utilized in experiments when cell confluency in culture vessel reached 70–80%. Cell lines were not utilized past passage 25.

### Patient samples

Tissue was acquired from HNSCC patients undergoing medically-indicated surgeries with written informed consent and approval of the University of Pennsylvania Institutional Review Board. HNSCC tumor and contralateral normal tissue were collected in TRIzol (Thermo Fisher Scientific, Waltham, MA, USA) from patients undergoing medically-necessary diagnostic biopsies or ablative surgeries.

### Data analyses and statistics

Raw data points in all bar graphs represents independent experiments with center representing the mean and error bars representing SEM. Responses were normally distributed by D’Agostino–Pearson test. We found no significant differences between variances of compared groups (F test for variance). Our ability to detect significant differences was estimated based on preliminary data using PS software (Vanderbilt Biostatistics) for independent comparisons. For most experiments, standard deviations of responses were determined to be ~10–15% of mean. To be 80% powered to see a 25% reduction (*p* < 0.05 considered significant), 4–6 samples were needed per comparison group. To be 80% powered to see a 50% difference, 3–4 samples were needed per group. Data were analyzed using *t-*test (two comparisons only, paired or unpaired t-tests as appropriate) or one-way ANOVA (>2 comparisons; Bonferroni and Dunnett’s posttests) in GraphPad Prism (San Diego, CA, USA); *p* < 0.05 was be considered significant. TCGA analysis was performed using GEPIA2 [30]. All image analysis was done in ImageJ.

### Supplementary information

Supplementary Material

## Data Availability

Requests for resources or reagents should be directed to and will be fulfilled by Robert J. Lee (rjl@pennmedicine.upenn.edu).

## Material availability

The published article includes all datasets generated or analyzed during this study. Raw numerical values used to generate bar graphs or traces are available upon request to Robert J. Lee (rjl@pennmedicine.upenn.edu).
