# Supplementary material for: GLUT1 inhibitor BAY-876 induces apoptosis and enhances anti-cancer effects of bitter receptor agonists in head and neck squamous carcinoma cells
Source: Cell Death Discov. 2024 Jul 25;10:339. doi: 10.1038/s41420-024-02106-z (PMC11282258; doi:10.1038/s41420-024-02106-z)
Supplement: Supplementary file 1 — Supplementary Material [file 41420_2024_2106_MOESM1_ESM.pdf]

SUPPLEMENTAL METHODS

Pharmacological Agents and Stock Solutions

BAY-876 (Cayman Chemicals) was dissolved in dimethyl sulfoxide (DMSO) at 100 mM. All subsequent experiments used BAY-876 at 100 µM or less, resulting in ≤ 0.1% DMSO in all experimental samples. WZB-117 (Cayman Chemicals) was dissolved and utilized in the same way. Bitter agonists lidocaine (Cayman Chemicals) and denatonium benzoate (TCI Chemicals) were dissolved at in Corning DMEM (with or without phenol depending on assay) at 20 mM. Lidocaine and denatonium were used at 5 and 1 mM, respectively. When used with BAY-876, lidocaine and denatonium were diluted to appropriate volume with desired concentration. BAY-876 was then added to volume for desired concentration. Other regents and source information are shown in Supplemental Table 2.

Computational Gene Expression Profiling using Depmap and TCGA

SLC2A (glucose transporter) expression was mapped using the Cancer Dependency Map Project (depmap; Broad Institute) in 62 total HNSCC immortalized cell lines. Pearson correlation method was utilized to determine expression from Expression Public 24Q2 portal data from a subset cell line (head and neck). Transcripts per million were graphed for each *SLC2A* gene for each individual cell line within subset.

SLC2A family genes were also analyzed using GEPIA2 (1). Individual cancer and non-cancer samples were evaluated for *SLC2A* expression. GEPIA2 was generated using HNSC (head and neck squamous cell carcinomas) from The Cancer Genome Atlas (TCGA) database. SLC2A expression was displayed as boxplot showing transcripts per million in log scale, with each dot representing individual patient samples. Significance determined by one-way ANOVA with $P < 0.01$ (*). *SLC2A1* expression was also graphed according to stage (I, II, III, IVA, IVB, or IVC) as volcano plot showing transcripts per million in log scale. Significance determined by one-way

ANOVA. Expression of all *SLC2A* family genes were compared amongst all cancer sets available from TCGA (tumor only). Data displayed as heat map showing transcripts per million in log scale.

Inflammatory cytokine expression in HNSCCs was generated using same method above for *SLC2A* individual boxplots.

Quantitative reverse transcription PCR (qPCR)

Cells were harvested and lysed in TRIzol when culture reached 70% confluency for measures of endogenous activity or after treatment period with pharmacological agent. Patient samples were digested in TRIzol for an hour and physically disbanded with RNase-Free Disposable Pellet Pestle (ThermoFisher) until tissue was fully lysed. RNA was isolated and purified using product protocol (Direct-zol RNA kit; Zymo Research). Final RNA was eluted in 25-30 µL of nuclease free water. RNA concentration of samples was measured using Tecan (Spark 10M; Mannedorf, Switzerland) NanoQuant plate. Between 200 – 500 ng of RNA were used to transcribe cDNA using MultiScribe Reverse Transcriptase (High-Capacity cDNA Kit; Thermo Fisher Scientific). A PCR Thermocycler was used to synthesize cDNA in one cycle of 25 °C for 10 minutes, 37 °C for 120 minutes, and 85 °C for 5 minutes. Samples were diluted with nuclease free water to 5 ng cDNA per µL (assuming ng of RNA in rxn = ng of cDNA synthesized). Gene expression in cDNA samples were quantified using TaqMan qPCR probes for *SLC2A1, SLC2A2, SLC2A6, SLC2A8, SLC2A9, SLC2A12, CXCL8* and *UBC* (Thermo Fisher Scientific). 2.5 µL of Fast Advanced, 0.25 µL of Taqman qPCR probes, and 0.25 µL of Nuclease free water was used per well (384-well qPCR plate) in combination of 2 µL of cDNA (at 5 ng/µL). This was done in triplicate for each sample. Gene expression was measure using QuantStudio 5 Real-Time PCR System for Human Identification during 40 cycles of 50 °C for 2 minutes , 95 °C for 2 minutes, 95 °C for 1 second, 60 °C for 20 seconds. Gene expression was calculate using *UBC* as an endogenous control due to stable expression in cancer cells (2). Cycle threshold (Ct) values were

averaged from triplicate wells for each sample (3). Fold-differences in gene expression were calculate using $2^{-\Delta\Delta CT}$ method:

$$\Delta\text{Ct (target gene)} = \text{Ct (target gene)}\ experimental\ sample - Ct(target\ gene)\ control\ sample$$

$$\Delta\text{Ct (UBC)} = \text{Ct (UBC)}\ experimental\ sample - Ct(UBC)\ control\ sample$$

$$\Delta\Delta\text{Ct} = \Delta\text{Ct (target gene)} - \Delta\text{Ct (UBC)}$$

$$Fold\ Change = 2^{-(\Delta\Delta\text{Ct})}$$

For endogenous/baseline gene expression (no experimental manipulation), the following calculations were used:

$$\Delta\text{Ct} = \text{Ct}\ (target\ gene)\ sample\ 1 - Ct(UBC)\ sample\ 1$$

$$\text{Relative expression (normalized to UBC)} = 2^{-(\Delta\text{Ct})}$$

Glucose Uptake Assays

A Glucose Colorimetric Assay Kit (Cayman) was used to measure glucose content in cell medium. Cells were treated with 200 µL +/- BAY-876 in a 96 well plate in phenol-free DMEM (Gibco). After treatment period, 100 µL of media was collected and used according to assay protocol (Cayman). Absorbance was measured using Tecan Spark 10 M. Experimental absorbance values were normalized to control.

pcDNA3.1 FLII12Pglu-700uΔ6 (gift from Wolf Frommer; Addgene plasmid #17866) was used to fluorescently label intracellular glucose (4). The plasmid was transfected into cells 24 hours prior to imaging with Lipofectamine 3000 (ThermoFisher Scientific). Each well of a 8-well glass chamber slide received 0.5 µg plasmid DNA, 1 µL p3000, and 0.5 µL Lipofectamine 3000 in 25 µL Optimem and 225 µL DMEM. Incubation of DNA and reagents prior to addition to cells was followed according to ThermoFisher Lipofectamine 3000 protocol. 1 hour prior to imaging, cells were glucose starved in Hank's Balanced Salt Solution (HBSS) (20 mM HEPES; pH 7.4; 1.8 mM Ca^{2+}, no glucose) +/- BAY-876. Cells' response to 25 mM glucose in HBSS imaged on

Olympus IX-83 microscope (20x 0.75 NA PlanApo objective), CFP/YFP filters (Chroma 89002-ET-ECFP/ EYFP) in excitation and emission filter wheels (Sutter Lambda LS), Orca Flash 4.0 sCMOS camera (Hamamatsu, Tokyo, Japan), Meta-Fluor (Molecular Devices, Sunnyvale, CA USA), and XCite 120 LED Boost (Excelitas Technologies). Change in fluorescence was calculated by subtracting background (area of no cells within frame) from the average intensity CFP-YFP (FRET) and YFP wavelengths in regions of interest. The ratio of CFP-YFP/YFP from each experimental repeat was normalized to value at time 0 of the recording. Peak fluorescence response and fluorescence response over time were graphed.

Fluorescent analog 2-NBDG (2-(N-(7-Nitrobenz-2-ox-1,3-diazol-4-yL)Amino) was used to measure glucose uptake. Cells were glucose starved +/- BAY-876 in HBSS (no glucose) for 1 hour prior to imaging. Glucose was replenished (HBSS + 25 mM glucose) in parallel with 2-NBDG (5) using the protocol from Cayman Chemical Glucose Uptake Kit (#600470). Images were acquired using FITC filters on Olympus imaging system described above. Fluorescence was quantified by subtracting background (area of no cells within frame) from average intensity of FITC wavelength in regions of interest. Normalized fluorescence values were graphed.

To measure NADH, cells were glucose starved for 1 hour +/- BAY-876 in HBSS (no glucose) prior to imaging . Protocol was followed as previously described (6). NADH is absorbs light at 340 ± 30 nm (without additional reagents). Cells were excited at 359 nm (~500 ms exposure) and fluorescence intensity was collected at 461 nm using DAPI Filter on Olympus imaging system described above. Fluorescence was measured with the addition of 25 mM glucose in HBSS. Fluorescence was quantified by subtracting background (area of no cells within frame) from average intensity of DAPI wavelength in regions of interest. Peak response and response over time were graphed.

Immunofluorescence

Cells were fixed on glass 8-well chamber slides with 4% formaldehyde in PBS $+Ca^{2+}/Mg^{2+}$ or with cold 100% methanol on ice (Na^{+}/K^{+} pump antibody only) for 20 (RT) or 3 minutes (on ice), respectively. Cells were washed 3x with PBS $+Ca^{2+}/Mg^{2+}$ with 5 minutes between each wash after each step described from this point on. Cells were blocked and permeabilized in buffer containing PBS with Ca^{2+} and Mg^{2+}, 0.2% saponin, 3% normal donkey serum, 1% BSA (blocking buffer) and 0.1% Triton X-100 at 4 °C for 30 minutes. Cells were incubated in primary antibody in blocking buffer (at either 1:100 or 1:200 dilution) overnight at 4 °C. Cells were incubated with secondary antibody in blocking buffer(1:500) for 2 hours at 4 °C in the dark. Cells were stained with AlexaFluor 488 phalloidin (actin) at 1:400 dilution in blocking buffer for 20 minutes at 4 °C in the dark. DAPI mounting medium (Abcam) was added (4 drops from bottle to cover cells) to label nuclei.

Patient tumor slices were cultured as described (7) and fixed using above described protocol. Images were taken on the Olympus IX83 system described above on a 60x 1.4N.A. oil objective. Fluorescence intensity was adjusted using HiLo look-up table (LUT) to best show observed results. Intensity values were set equally across all compared images. Images were assigned colored LUTs and cropped to show region of interest. Scale bars range from 20 – 50 uM as indicated. No further processing was done.

Cell Viability

Cells were treated for 24 or 48 hours +/- BAY-876 or for 24 hours +/- BAY-876 with lidocaine or denatonium benzoate. Treatment was removed and cells were washed 1x with PBS. Crystal violet 0.1% in deionized water) was used to stain remaining adherent, viable cells (200 µL per well in 48 well plate). Crystal violet stayed on cells for 5 minutes for uptake of stain . Crystal violet was aspirated and remaining stains were washed 3x with deionized water and left to dry for 24 hours. Crystal violet stains were then dissolved with 30% acetic acid in deionized water.

Absorbance at 590 nm measured on a Tecan (Spark 10M; Mannedorf, Switzerland). Absorbance values for experimental wells were normalized to control (no treatment).

XTT assay was done according to ThermoFisher protocol to measure NADH production. 30 µL of XTT dye (stock 0.1 mg/uL in DMSO) and 7.5 µL phenazine methosulfate (stock 3 mg/mL in PBS) (MilliporeSigma) were added to 3 mL of phenol-free DMEM. Cells (in 48 well plate) were washed 3x with PBS and treated at 1.25x normal concentration in phenol-free DMEM (200 uL per well). 50 µL of XTT/phenazine methosulfate solution was added to each well (adjusting final treatment concentration to 1x). Absorbance values were measured at 475 and 660 nm over 6 hours every 10 minutes on Tecan Spark 10M. Final values were calculated by subtracting 660 nm wavelength from 475 nm wavelength and normalizing to control well(s).

Ratiometric, mitochondrial membrane potential indicator dye, JC-1, was loaded into cells on a glass-bottom at 1 µg/mL (stock 1 mg/mL in DMSO) in phenol-free DMEM. Cells were loaded for 15 minutes in the dark at RT. Dye was aspirated and cells were washed 3x with PBS. BAY-876 treatment was added to each appropriate well. Fluorescence was measured on Tecan Spark 10 M on FITC and TRITC filters every 15 minutes over 12 hours at 37 °C, 5% CO_2. Ratio of FITC fluorescence intensity to TRITC fluorescence intensity was normalized to time 0 and plotted over time, with an increase in normalized ratio representing a decreased in mitochondrial membrane potential.

FaDu cells were trypsonized into single-cell suspension and plated on on low-attachment 96-well plate in DMEM/F-12 1:1 media supplemented with 10 ng/µL epidermal growth factor (EGF) and fibroblast growth factor (FGF) and 1% B-17 Supplemental (8). Spheroids were cultured for 2 days before treatment with BAY-876. After 24 hours, live cell DIC images were taken on Olympus IX-83 microscope system. In addition, FaDu cells were plated (as described above) and co-treated with BAY-876 +/- lidocaine or denatonium to observe initial spheroid formation. Spheroid formation was imaged at 24 hours.

CellEvent

CellEvent Green Caspase 3/7 dye (ThermoFisher Scientific) was added to cells in a glass bottom plate with BAY-876 alone or with BAY-876 +/- lidocaine or denatonium benzoate in phenol-free DMEM. For every 500 µL of phenol-free DMEM treatment, 1 drop of CellEvent reagent was used (dropper bottle). Representative images taken with FITC filter on Olympus IX-83 microscope using 20x objective. Quantitative fluorescence values measured on a Tecan Spark 10M over 16 hours at 37 °C, 5% CO_2. Fluorescence intensity adjusted using the HiLo look-up table (LUT) to best show observed results. Intensity values were set equally across all images compared or normalized to control for quantification.

Cytokine Inhibition Assay

Cells were treated with 0.1 µg of TNF-alpha in 2 mL of DMEM +/- 100 µM BAY-876 for 24 hours. *CXCL8* was measured using qPCR method described above.

SUPPLEMENTAL FIGURES

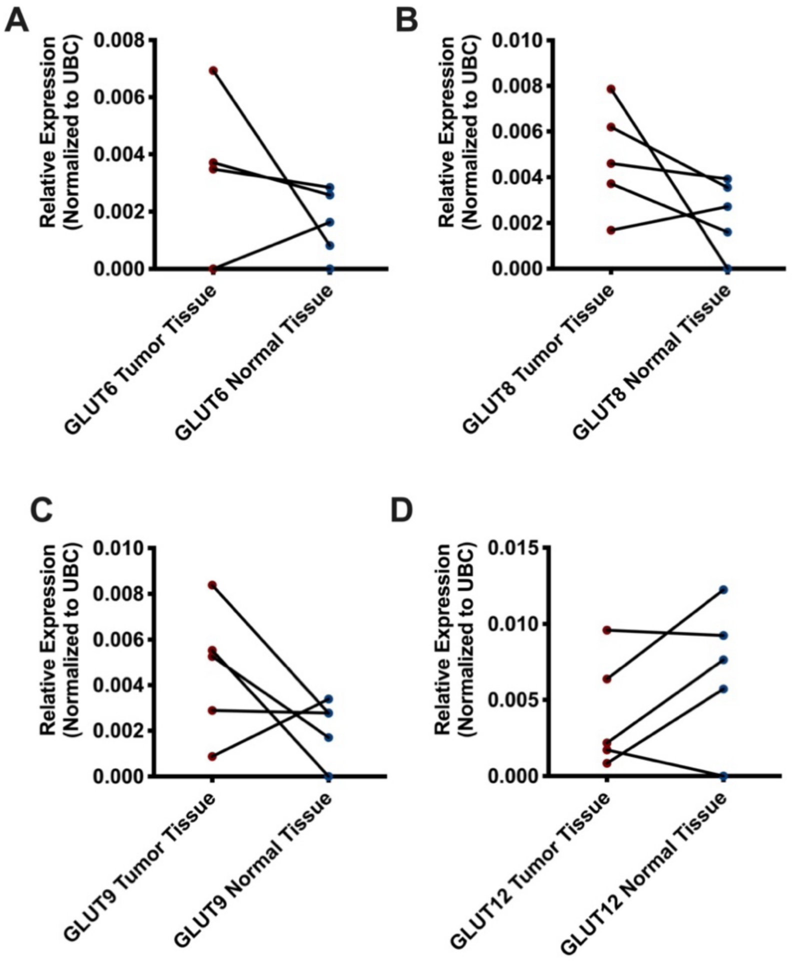

Supplemental Figure 1. Glucose transporters are expressed in HNSCC patient samples. A-D) Matched RNA expression of *A) SLC2A6 (*GLUT6), *B) SLC2A8* (GLUT8), *C) SLC2A9* (GLUT9), and *D) SLC2A12* (GLUT12) between HNSCC individual patients' tumor and normal tissue. Expression is relative to endogenous UBC. Expression means ± SD with n=6 patients. No significant difference by paired t-test between the tumor and normal tissue. P < 0.05 (*), P < 0.01 (**), P < 0.001 (***), and no statistical significance (ns or no indication).

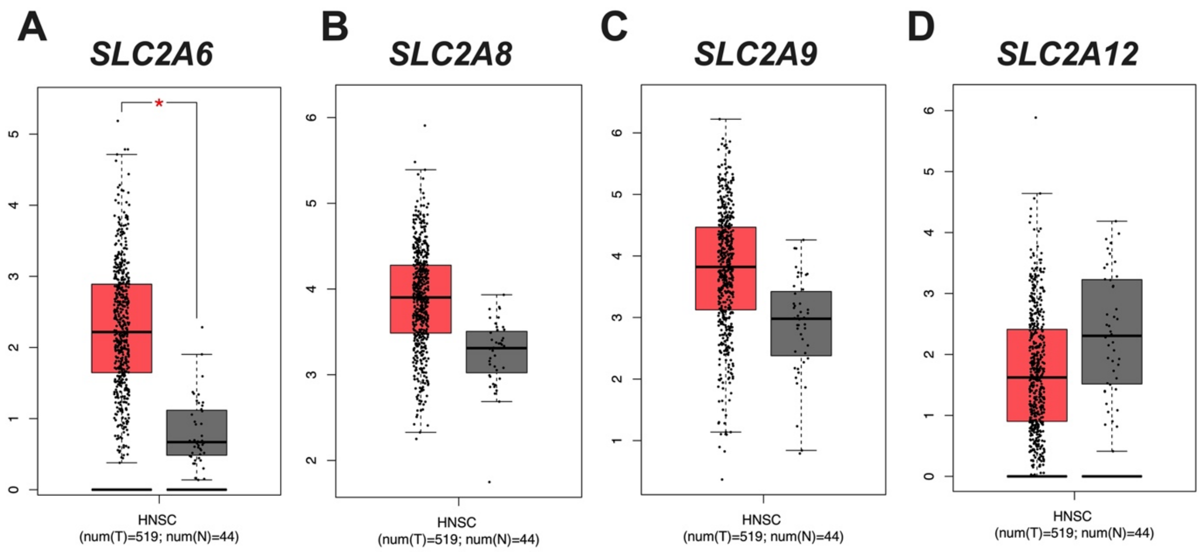

Supplemental Figure 2. *SLC2A* GLUTs are expressed in HNSCC. A-D) Boxplot of *A) SLC2A6* (GLUT6), *B) SLC2A8* (GLUT8), *C) SLC2A9* (GLUT9), and *D) SLC2A12* (GLUT12) expression in HNSCC tumor samples (n = 519) and normal tissue samples (n = 44), generated using GEPIA2 (1).

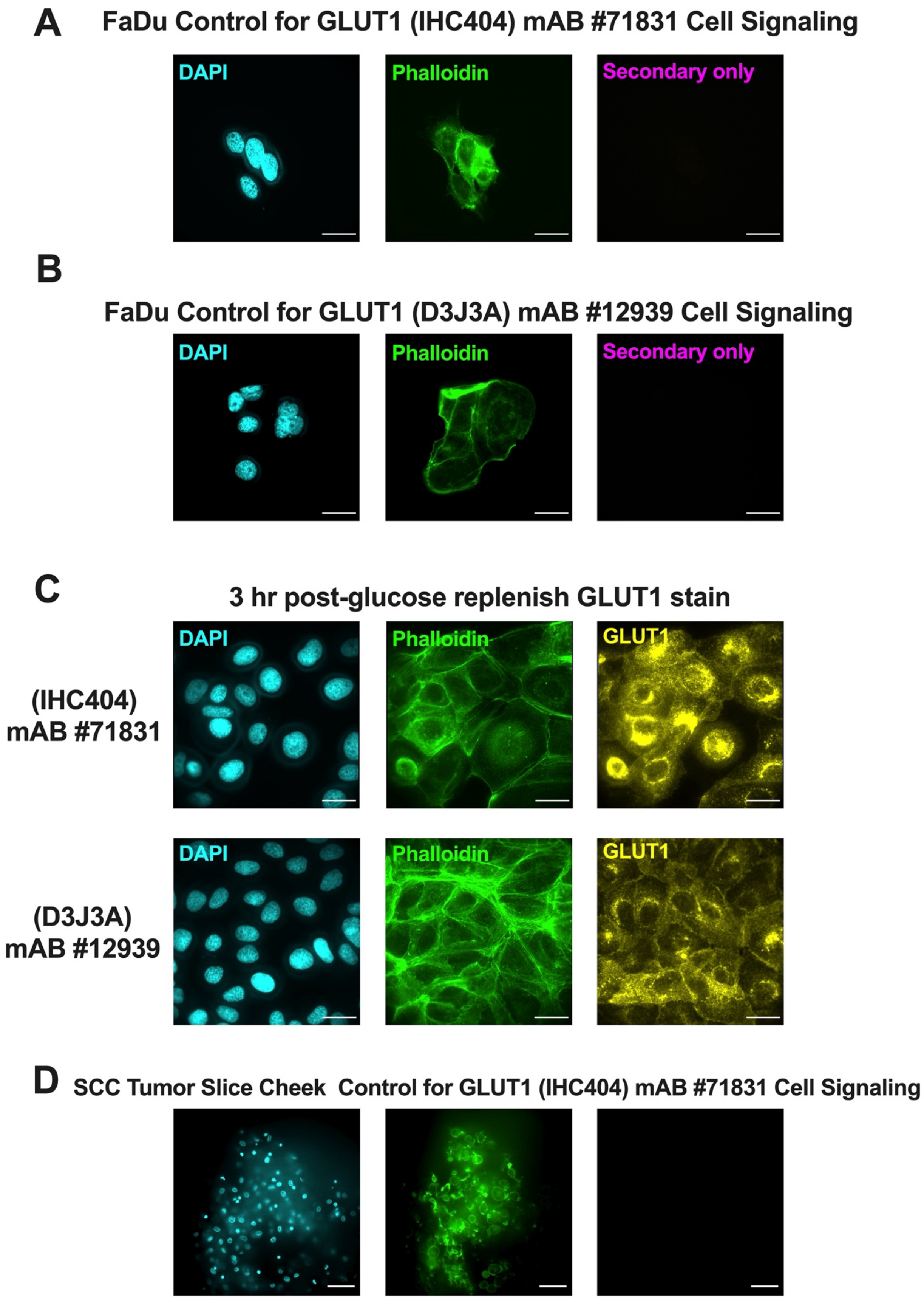
A
FaDu Control for GLUT1 (IHC404) mAB #71831 Cell Signaling
DAPI
Phalloidin
Secondary only
B
FaDu Control for GLUT1 (D3J3A) mAB #12939 Cell Signaling
DAPI
Phalloidin
Secondary only
C
3 hr post-glucose replenish GLUT1 stain
(IHC404) mAB #71831
DAPI
Phalloidin
GLUT1
(D3J3A) mAB #12939
DAPI
Phalloidin
GLUT1
D
SCC Tumor Slice Cheek Control for GLUT1 (IHC404) mAB #71831 Cell Signaling

Supplemental Figure 3. GLUT1 localization is not dependent on extracellular glucose levels. A & B) GLUT1 expression with no primary antibody in FaDu cells from immunofluorescence experiments using *A)* mAB #71831 or *B)* mAB#12939 antibodies from Cell Signaling with DAPI (cyan, nucleus) and phalloidin (green, actin) markers. **C)** GLUT1 expression in SCC47 cells after replacing DMEM high glucose media (4.5 g/L) 3 hours before fixing. Both antibodies were used to confirm the localization of GLUT1. Cells were fixed with 4% formaldehyde, blocked and permeabilized, and stained with 1:200 primary antibody for 4 hours. AlexaFluor 555 or 647 goat anti-mouse or -rabbit were used as secondary antibodies at 1:500 for 2 hours. Cells were imaged on Olympus Live Cell Imaging System with oil immersion 60x objective. Scale bars = 30 μm. Images best represent phenotype observed in n>12 separate cultures.

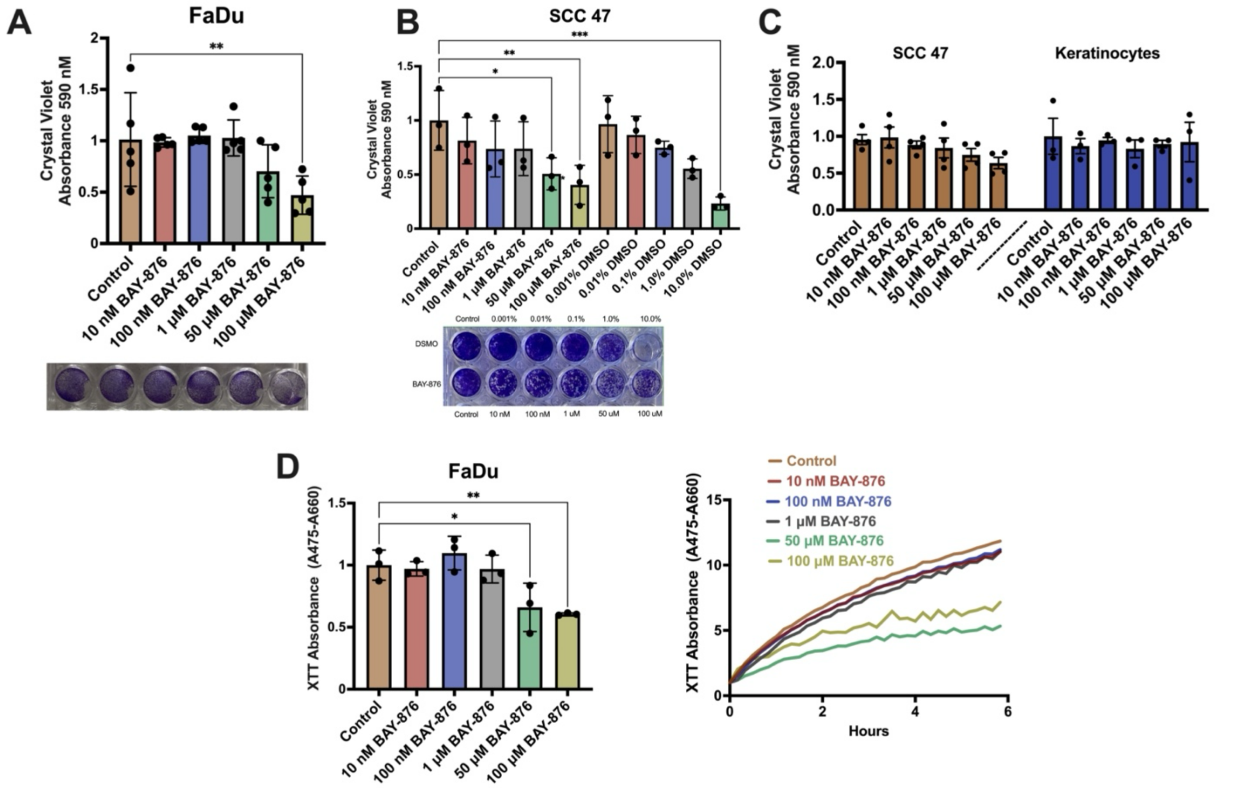

Supplemental Figure 4. BAY-876 decreases viability and cell metabolism. A) Absorbance values of crystal violet stain representing alive/adherent FaDu cells treated with BAY-876 over 24 hours. Absorbance mean ± SD with n=5 of separate passages of cells. Significance determined by 1-way ANOVA with Bonferroni’s posttest comparing BAY-876 treatments to control. **B)** Absorbance values of crystal violet stain representing alive/adherent SCC47 cells treated with BAY-876 or DMSO over 24 hours. Note that the highest concentration of DMSO vehicle in the BAY-876 treated samples is 0.1% DMSO with 100 μM BAY-876. DMSO at 0.1% alone had no effects on cell viability. Absorbance mean ± SD with n=3 of separate passages of cells. Significance determined by 1-way ANOVA with Bonferroni’s posttest comparing BAY-876 or DMSO treatments to control. **C)** Absorbance values of crystal violet stain representing alive/adherent SCC47 cells or oral keratinocytes treated with BAY-876 over 24 hours. Absorbance mean ± SD n=4 of separate passages of cells. Insignificant results determined by paired t-tests between each equivalent treatment of BAY-876 between cell types. **D)** Absorbance values of NADH production (via addition of XTT dye) at 6 hours (left) and over time (right) in FaDu cells. A decrease in absorbance (475 nm – 660 nm) indicated reduced NADH production. Absorbance means ± SD with n = 3 separate cell passages. Significance determined by 1-way ANOVA with Bonferroni’s posttest comparing BAY-876 to control. $P < 0.05$ (*), $P < 0.01$ (**), $P < 0.001$ (***), and no statistical significance (ns or no indication).

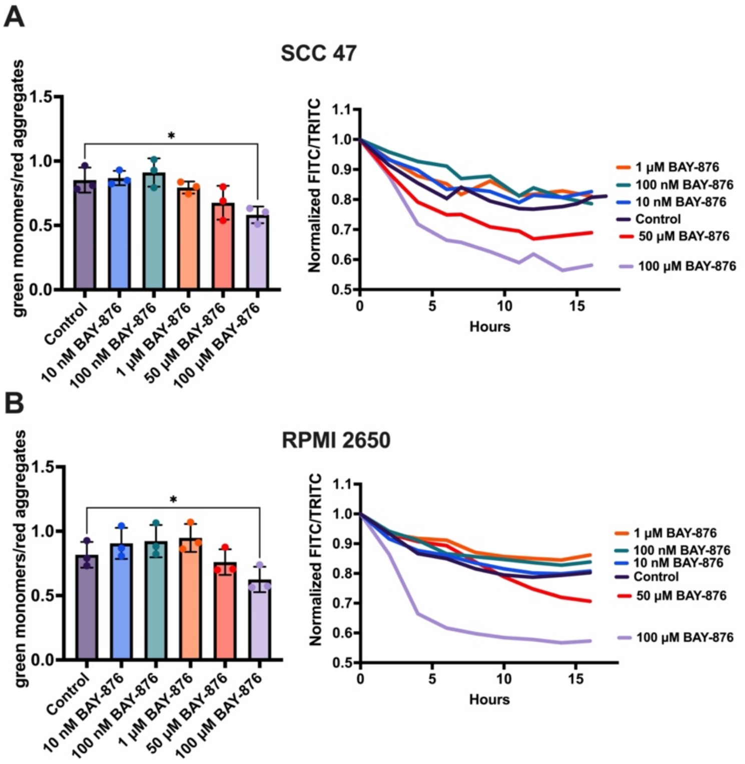

Supplemental Figure 5. BAY-876 hyperpolarizes mitochondria in HNSCC cells. A & B) Ratiometric fluorescence values of JC-1 green dye monomers (representing low mitochondrial potential) to red dye aggregates (representing high mitochondrial potential) of A) SCC 47 and B) RPMI 2650 cells at 16 hours (left bar graph) and over time (right xy graph). Fluorescence means ± SD with n = 3 separate cell passages. Significance determined by 1-way ANOVA with Bonferroni's posttest comparing BAY-876 to control. $P < 0.05$ (*), $P < 0.01$ (**), $P < 0.001$ (***), and no statistical significance (ns or no indication).

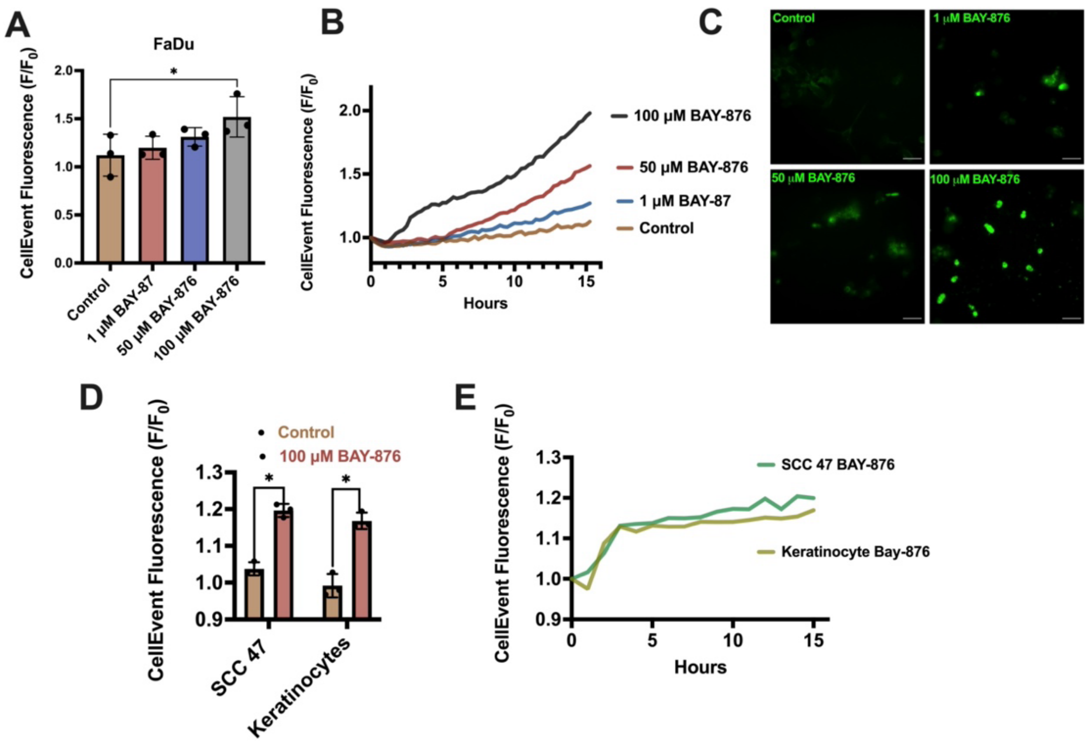

Supplemental Figure 6. BAY-876 induces apoptosis in HNSCC cells. A-B) Fluorescence values and images of CellEvent caspase-3 and -7 dye (increase in fluorescence indicative of caspase activation) at 15 hours (left bar graph and representative images) and over time (middle xy plot) of FaDu cells treated with BAY-876. Fluorescence means ± SD with n = 3 separate cell passages. Significance determined by 1-way ANOVA with Bonferroni’s posttest comparing BAY-876 to control. Fluorescence values and images of CellEvent caspase-3 and -7 dye (increase in fluorescence indicative of caspase activation) at 15 hours (left bar graph) and over time (right xy plot) of *SCC47* cells or oral keratinocytes treated with BAY-876. Fluorescence means ± SD with n = 3 separate cell passages. Significance determined by 1-way ANOVA with Bonferroni’s posttest comparing BAY-876 to control. P < 0.05 (*), P < 0.01 (**), P < 0.001 (***), and no statistical significance (ns or no indication).

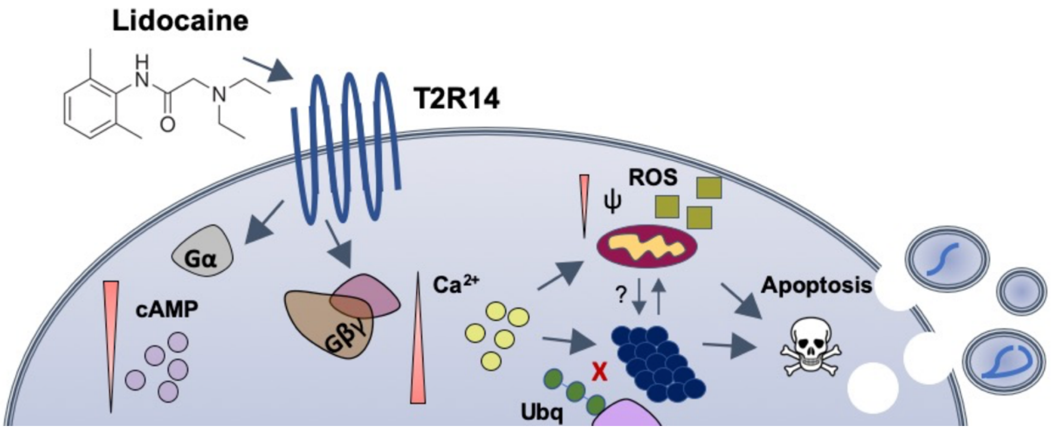

Supplemental Figure 7. Mechanisms of bitter taste receptor (T2R)-induced cell death. As described and referenced in the main text, activation of T2Rs (shown with lidocaine activating T2R14) results in both a Gα-induced cAMP decrease as well as a Gβγ-induced Ca^{2+} influx into the mitochondria causes excess reactive oxygen species (ROS) production that results in inhibition of the ubiquitin (Ubq) proteasome system and activation of apoptosis.

SUPPLEMENTAL TABLES

Supplemental Table 1. Summary of effects of BAY-876 on HNSCC cellular health.

Experimental Assay	Figure(s)	Cell Line(s)	Result
**Colorimetric Glucose Uptake Assay**	4B	SCC47	Decrease in glucose uptake over 24 hours
**Crystal Violet**	5A & B; S4A	SCC47 RPMI2650 FaDu	Decrease in cell viability over 24 hours
**XTT**	5D & E; S4D	SCC47 RPMI2650 FaDu	Decrease in cell metabolism (NADH) over 6 hours
**JC-1**	S5	SCC47 RPMI2650	Increase in mitochondrial membrane potential over 15 hours
**CellEvent**	6A-F; S6A-C	SCC47 RPMI2650 FaDu	Increase in caspase-3 and -7 cleavage over 15 hours
**Crystal Violet with Bitter Agonists**	7C-H	SCC47	Further decrease in cell viability over 24 hours with lower concentrations of BAY-876 and bitter agonists in combination
**CellEvent with Bitter Agonists**	7I	SCC47	Further increase in caspase-3 and -7 cleavage over 15 hours with lower concentrations of BAY-876 and bitter agonists in combination

Supplemental Table 2. Key reagents and resources used in this study.

REAGENT OR RESOURCE	SOURCE	IDENTIFIER
**Antibodies**		
Anti-GLUT1 (IHC404)	Cell Signaling	71831
Anti-GLUT1 (D3J3A)	Cell Signaling	12939
Anti-TAS2R14/TRB1	LS Bio	C386266
Anti-Na^+/K^+-ATPase	Abcam	ab76030
Anti-GM130	BD Trans. Laboratories	610822
Anti-LAMP1	Abcam	ab24170
Anti-rabbit AlexaFluor 647	Life Technologies	A31537
Anti-rabbit AlexaFluor 555	Life Technologies	A31851
Anti-mouse AlexaFluor 555	Life Technologies	A31570
**Chemicals & Reagents**		
DMSO (Dimethyl sulfoxide)	Millipore Sigma	276855
BAY-876	Cayman Chemical	19961
WZB-117	Cayman Chemical	19900
Lidocaine	Cayman Chemical	20081
Denatonium Benzoate	TCI Chemicals	D2124
Saponin	Millipore Sigma	S7900
Bovine Serum Albumin	Millipore Sigma	A2153
Crystal Violet	Millipore Sigma	C0775-25G
JC-1	ThermoFisher	T3168
CellEvent™ Caspase-3/7 Detection Reagent	ThermoFisher	C10423
XTT (sodium 3´-(1- (phenylaminocarbonyl)-3,4- tetrazolium)-bis (4-methoxy6-nitro) benzene sulfonic acid hydrate	ThermoFisher	X6493
Lipofectamine 3000	ThermoFisher	L3000075
TriZol	ThermoFisher	15596026
TaqMan Fast Advanced Master Mix	ThermoFisher	4444946
Normal Donkey Serum	Abcam	ab7475
Fluoroshield Mounting Medium w/ DAPI	Abcam	Ab104131
Phalloidin AlexaFluor 488	Life Technologies	A12379
Formaldehyde	Millipore Sigma	47608
Triton X-100	Cayman Chemicals	600217
Mitotracker	ThermoFisher	M22425
Cell-Based Assay NBD Glucose	Cayman Chemicals	600471
D-(+)-Glucose	ThermoFisher	A16828.36
Sucrose	ThermoFisher	A15583.36
TNF-alpha	GenScript	Z02682-10
Hank's Balanced Salt Solution (HBSS)	In House	
PBS	In House	
PBS with Ca^{2+} & Mg^{2+}	In House	
UltraPure Nuclease Free Distilled Water	ThermoFisher	10977015
**Critical Commercial Assays**		
High-Capacity cDNA Reverse Transcription Kit	ThermoFisher	4368814
Direct-zol RNA Miniprep	Zymo Research	R2051
Glucose Colorimetric Assay Kit	Cayman Chemical	10009582

**Experimental Models**		
SCC90	ATCC	CRL-3239
UMSCC47 (SCC47)	Millipore Sigma	SCC071
FaDu	ATCC	HTB-43
RPMI2650	ATCC	CCL-30
Primary Gingival Keratinocytes	ATCC	PCS-200-014
**Cell Culture Reagents**		
Corning DMEM 1x	ThermoFisher	MT10013CV
Gibco DMEM no phenol red	ThermoFisher	31-053-028
DMEM/F-12	ThermoFisher	11320033
Epidermal Growth Factor	Sigma-Aldrich	SRP3027-500UG
Corning Basic Fibroblast Growth Factors	Life Sciences	354060
B-27 Supplemental (minus Vitamin A)	ThermoFisher	12587010
Dermal Cell Basal Medium	ATCC	PCS-200-030
Keratinocyte Growth Kit	ATCC	PCS-200-040
OptiMEM I (1x) Reduced Serum Medium	ThermoFisher	31985062
GenClone Fetal Bovine Serum	Genesee Scientific	25-514
Gibco Penicillin-Streptomycin-Glutamine	ThermoFisher	10-378-016
MEM Non-Essential Amino Acids	ThermoFisher	11140050
Trypsin-EDTA (0.25%), phenol red	ThermoFisher	25200056
**Oligonucleotides**		
Taqman Assay for SLC2A1	ThermoFisher	Hs00892681_m1
Taqman Assay for SLC2A2	ThermoFisher	Hs01096908_m1
Taqman Assay for SLC2A6	ThermoFisher	Hs00214042_m1
Taqman Assay for SLC2A8	ThermoFisher	Hs00205863_m1
Taqman Assay for SLC2A9	ThermoFisher	Hs01119178_m1
Taqman Assay for SLC2A12	ThermoFisher	Hs00376943_m1
Taqman Assay for CXCL8	ThermoFisher	Hs00174193_m1
Taqman Assay for UBC	ThermoFisher	Hs01871556_s1
**Recombinant DNA**		
pcDNA3.1_FL1112Pglu700Δ6	Wolf Frommer	Addgene 17866
**Software and Algorithms**		
MetaFluor	Molecular Devices	N/A
MetaMorph	Molecular Devices	N/A
QuantStudio 5	Applied Biosystems, Inc	N/A
Prism v9	GraphPad Software	N/A
ImageJ/FIJI	Open Source	N/A
**Databases**		
GEPIA2	Open Source	N/A
Cancer Dependency Map (depmap)	Open Source	N/A

Supplemental Table 3. Pharmacological agonists and inhibitors used in this study. Sources are shown in Supplmental Table 2.

**Agonist or inhibitor**	**Target**
BAY-876	GLUT1 inhibitor
WZB-11	GLUT1 inhibitor
Lidocaine	T2R14 agonist
Denatonium benzoate	T2R4 agonist
TNFα	TNFR1/2 agonist

Supplemental References

1. Tang Z, Kang B, Li C, Chen T, Zhang Z. GEPIA2: an enhanced web server for large-scale expression profiling and interactive analysis. Nucleic Acids Res. 2019;47(W1):W556-W60.

2. Christensen JN, Schmidt H, Steiniche T, Madsen M. Identification of robust reference genes for studies of gene expression in FFPE melanoma samples and melanoma cell lines. Melanoma Res. 2020;30(1):26-38.

3. Schmittgen TD, Livak KJ. Analyzing real-time PCR data by the comparative C(T) method. Nat Protoc. 2008;3(6):1101-8.

4. Takanaga H, Chaudhuri B, Frommer WB. GLUT1 and GLUT9 as major contributors to glucose influx in HepG2 cells identified by a high sensitivity intramolecular FRET glucose sensor. Biochim Biophys Acta. 2008;1778(4):1091-9.

5. Zou C, Wang Y, Shen Z. 2-NBDG as a fluorescent indicator for direct glucose uptake measurement. J Biochem Biophys Methods. 2005;64(3):207-15.

6. Wang HW, Wei YH, Guo HW. Reduced nicotinamide adenine dinucleotide (NADH) fluorescence for the detection of cell death. Anticancer Agents Med Chem. 2009;9(9):1012-7.

7. Runge A, Mayr M, Schwaiger T, Sprung S, Chetta P, Gottfried T, et al. Patient-derived head and neck tumor slice cultures: a versatile tool to study oncolytic virus action. Sci Rep. 2022;12(1):15334.

8. Kadletz L, Heiduschka G, Domayer J, Schmid R, Enzenhofer E, Thurnher D. Evaluation of spheroid head and neck squamous cell carcinoma cell models in comparison to monolayer cultures. Oncol Lett. 2015;10(3):1281-6.
